# Does it matter who addresses whom in vaccination promotion campaign?

**DOI:** 10.1093/eurpub/ckac129.737

**Published:** 2022-10-25

**Authors:** S Cvjetkovic, V Jeremic- Stojkovic, S Mandic-Rajcevic, J Jankovic

**Affiliations:** 1 Department of Humanities, Faculty of Medicine, University of Belgrade, Belgrade, Serbia; 2 Institute of Social Medicine, Faculty of Medicine, University of Belgrade, Belgrade, Serbia

## Abstract

**Background:**

A vaccine promotion campaign is primarily grounded on the selected message features, namely, a carefully chosen information source. People holding diverse views towards vaccination could experience the same information source differently, and it is the comprehension of these diversities that is important to tailor effective interventions. The aim of this study was to determine differences in perceived source credibility between the vaccinated and unvaccinated.

**Methods:**

Overall 172 adults aged 18 and older from Western Balkans both vaccinated and unvaccinated, voluntarily after obtaining informed consent, were randomly assigned to one of four message interventions. The messages were developed combining two prototypical COVID-19 vaccine decision narratives (determined vs. hesitant) with two communication sources (physician vs. lay peer), resulting in four conditions: determined physician, hesitant physician, determined peer, hesitant peer. After the message exposure, participants evaluated three components of source credibility - expertise, trustworthiness and, goodwill. Two-way ANOVA was applied.

**Results:**

Compared to the vaccinated, the unvaccinated judged the source as less trustworthy (p < 0.01), regardless of the message they have been exposed to. Although not statistically significant (p = 0.064), the unvaccinated evaluated all sources with the exception of hesitant physician as having a lower level of good intentions. Vaccinated perceived the determined physician as a source with most expertise, while unvaccinated attributed highest expertise to the hesitant physician (without significant difference (p = 0.719)).

**Conclusions:**

The unvaccinated are generally less likely to experience the information sources as goodwill and trustworthy. In order to perceive the source as more competent the focus should be on the objective characteristics of the communicator, as well as on the congruency in attitudes between the communicator and the audience.

